# Lack of association between the CHL1 gene and adolescent idiopathic scoliosis susceptibility in Han Chinese: a case-control study

**DOI:** 10.1186/1471-2474-15-38

**Published:** 2014-02-10

**Authors:** Xu-sheng Qiu, Feng Lv, Ze-zhang Zhu, Bang-ping Qian, Bin Wang, Yang Yu, Yong Qiu

**Affiliations:** 1 Spine Surgery, Drum Tower Hospital, Nanjing University Medical School, Nanjing, China

**Keywords:** Adolescent idiopathic scoliosis, CHL1, Chinese, Polymorphism

## Abstract

**Background:**

A previous genome-wide association study (GWAS) suggested a strong association between the single nucleotide polymorphism (SNP) rs10510181 in the proximity of the gene encoding a cell adhesion molecule with homology to L1CAM (CHL1) and adolescent idiopathic scoliosis (AIS) in Caucasians. To clarify the role of *CHL1* in the etiopathogenesis of AIS, we performed a case-control replication study in a Han Chinese population.

**Methods:**

Five hundred female AIS patients between 10 and 18 years of age, as well as 500 age- and sex-matched controls were included. This study was conducted as a 2-stage case-control analysis: initial screening for the association between AIS and SNPs in and around the CHL1 gene (186 cases and 169 controls) followed by a confirmation test (314 cases and 331 controls). rs10510181 and 4 SNPs (rs2055314, rs331894, rs2272522, and rs2272524) in the CHL1 gene were selected for genotyping.

**Results:**

Putative associations were shown between AIS and rs10510181, rs2055314, and rs2272522 in stage I. However, the associations were not confirmed in stage II. For rs10510181, the genotype frequencies were GG 28.8%, GA 46.2%, and AA 25.0% in AIS patients and GG 29.8%, GA 48.8%, and AA 21.4% in controls. No significant difference was found in genotype distribution between cases and controls (P = 0.39). Similarly, the genotype and allele distribution were comparable between case and control for rs2055314 and rs2272522.

**Conclusions:**

There was no statistical association between polymorphisms of the CHL1 gene and idiopathic scoliosis in a Chinese population.

## Background

Adolescent idiopathic scoliosis (AIS) is a complex 3-dimensional deformity of the spine that occurs most commonly in girls during the peripubertal period [1]. Despite decades of research, its specific cause has not been determined [2]. To date, many factors have been proposed to be involved in its etiology, such as genetic factor, nervous system, skeletal growth, hormone, and metabolic dysfunction [3]. It has been strongly suggested that genetic factor plays an important role in the development and progression of AIS [4,5]. Many AIS susceptibility loci have been identified through genome-wide linkage analyses and genome-wide association study [6-8]. Several promising candidate genes for AIS susceptibility have been reported, including the genes for estrogen receptor [9], melatonin 1B receptor [10], matrilin-1 [11] and ladybird homeobox 1 [6]. However, to our knowledge, only few genes can be replicated for their association in different ethnic populations [12-14].

Recently, Sharma et al. [15] performed a genome-wide association study (GWAS) based on transmission-disequilibrium tests in 419 AIS families, which identified associated single nucleotide polymorphisms (SNPs) in the proximity of the cell adhesion molecule with homology to the L1CAM (CHL1) gene. They genotyped additional SNPs in chromosome 3p26.3 and tested the association in two follow-up case-control cohorts. They obtained the strongest association in rs10510181 with all three cohorts combined (P = 2.58 × 10^-8^). *CHL1* encodes an axon guidance protein related to Robo3 [16], mutations of which could lead to horizontal gaze palsy with progressive scoliosis (HGPPS), a rare disease marked by severe scoliosis [17]. To confirm the association between rs10510181 and AIS, and to explore whether the CHL1 gene is associated with the occurrence of AIS, we conducted the present genetic association study in a Han Chinese population.

## Methods

### Subjects

This study initially screened 186 AIS patients and 169 controls for the association between AIS and SNPs in and around the CHL1 gene. Subsequently, the sample size was enlarged to 500 AIS patients and 500 controls for further validation. All patients were ethnically Han Chinese females aged between 11 and 18 years who were seen at our spine center between 2006 and 2010. The diagnosis of AIS was confirmed by a standing X-ray film of the whole spine. The curvature magnitude was measured by Cobb’s method. Only patients with a Cobb angle larger than 20 degrees were included in this study. Patients with congenital scoliosis, neuromuscular scoliosis, scoliosis with skeletal dysplasia, scoliosis with known endocrine and connective tissue abnormalities, or prior treatment for scoliosis were excluded. The control age- and sex-matched adolescent Han Chinese girls were recruited from local secondary schools through health examinations. All controls were examined with either a forward bending test or a radiograph if necessary to rule out any spine deformities. Any potential evidence of bone diseases, metabolic diseases, growth disturbances and other diseases known to affect normal bone metabolism were excluded. Informed consents for DNA analysis were obtained from all subjects or their parents. The protocols and the procedure were approved by the Committee on Medical Ethics of Nanjing Drum Tower Hospital.

### Genotyping

The SNP rs10510181, the one that showed strongest association with AIS in the study by Sharma et al. [15], was selected for the analysis. Furthermore, four SNPs rs2055314, rs331894, rs2272522, and rs2272524 were selected from dbSNP (http://www.ncbi.nlm.nih.gov). These SNPs span the entire region around the CHL1 gene with average intervals of approximately 30.5 kb [18].

Total genomic DNA was extracted from peripheral blood samples using a DNA extraction kit (Promega, Madison, WI), following to the manufacturer’s instructions. Identification of the polymorphisms was performed using the polymerase chain reaction-restriction fragment length polymorphism (PCR-RFLP) method. The primers were listed in Table 1. PCR was carried out in a total volume of 20 μL, consisting of 10 μL of 2 × PCR mix, 0.2 μL of each oligonucleotide primer and 8.6 μL of sterile deionized H_2_O. A typical 35 amplification cycle consisted of 30 seconds at 96°C, 45 seconds at the annealing temperature, and 30 seconds at 72°C (Table 1). The final elongation step was 7 minutes at 72°C. For restriction enzyme digestion, PCR products were subjected to 3 to 5 U of the required enzyme in the presence of the accompanying buffer and incubated overnight. The digested PCR products were visualized using electrophoresis in 2% or 4% agarose gels. A total of 10% of the samples were tested twice to validate the genotyping results with a reproducibility of 100%.

**Table 1:** **Primers and conditions of PCR**-**RFLP analysis**

**SNPs**	**Primer Sequence 5**′-**3**′	**Annealing temperature (C)**	**PCR size (bp)**	**Enzyme**	**Restriction fragment size (bp)**
rs2272522	F:AAGAGGACTAATCGTATATCTAATGGTC R: TGAAATTCCATTAATAGGCACTGA	57	110	*AvaII*	C:25 + 85
					T:110
rs331894	F: CAGAAACGACCCAGTAGACTCC	59	113	*MnlI*	A:49 + 37 + 17 + 10
	R: GTTTTCTTGCCCTCCCTTTC				
					G:86 + 17 + 10
rs2055314	F: TCCATCCCATGCACATCTTA	59	492	*HindIII*	C:492
	R: TGTCCGTCCACACATGCTAT				
					T:227 + 245
rs2272524	F:AAGGTTTTCTTTGTATTTTACTTTGC	59	106	*HhaI*	G:27 + 79
	R: TCACCACCAATTTTGTTCCA				
					A:106

PCR-RFLP: polymerase chain reaction-restriction fragment length polymorphism.

### Statistical analysis

The statistical analyses were performed using the SPSS software (version 13.0, Chicago, USA). The differences in genotype distributions and allelic frequencies between AIS patients and controls were examined using the χ2 test. The odds ratios (OR) and 95% confidence interval (95% CI) ranges were calculated by using logistic regression. Significance was considered at a P-value <0.05. Sample size calculation was done using Quanto (Version 1.2.4; USC, Los Angeles, CA). 473 cases and controls are needed to detect the same OR (1.49) as previously reported for rs10510181 [15] with 80% power assuming MAF (minor allele frequency) of 10% for the log-additive model.

## Results

The mean age was 14.7 years for AIS patients and 14.6 years for the controls. No significant different was found for body height between the two groups. However, the body weight was significantly lower in AlS patients than in normal controls. The mean maximum Cobb angle was 33.8° in AIS patients (Table 2).

**Table 2:** Characteristics of two study groups

	**AIS**	**Control**	**P-value**
Age (years)	14.7 ± 1.8	14.6 ± 1.8	0.93
Female (%)	500 (100)	500 (100)	1
Han Chinese (%)	500 (100)	500 (100)	1
Height (cm)	159.8 ± 5.7	160.0 ± 6.0	0.64
Weight (kg)	45.7 ± 6.3	52.6 ± 8.7	<0.001
Maximum Cobb angle (º)	33.8 ± 12.4	-	-

AIS = adolescent idiopathic scoliosis.

A total of 1000 subjects (500 cases and 500 controls) were successfully genotyped and subjected to the statistical analysis. The distributions of the alleles and genotypes for the five SNPs in stages I and II were presented in Table 3 and Table 4. No significant deviation of genotype frequencies from the Hardy-Weinberg equilibrium was noted in the AIS and control groups.

**Table 3:** The genotype and allele frequencies of SNPs in and around the CHL1 gene in AIS in a Chinese Han population

**Genotype**	**AIS (%)**	**Control (%)**	**P-value (χ2)**	**Alleles**	**AIS (%)**	**Control (%)**	**P**-**value (χ2)**	**OR (95% CI)**
rs2272524								
CC	56(30.1)	43(25.4)		C	202(54.3)	169(50.0)		C *vs*. T
CT	90(48.4)	83(49.1)	0.53	T	170(45.7)	169(50.0)	0.25	1.12 (0.89-1.60)
TT	40(21.5)	43(25.4)						
rs331894								
GG	48(25.8)	39(23.1)		G	186(50.0)	170(50.3)		G *vs*. A
GA	90(48.4)	92(54.4)	0.52	A	186(50.0)	168(49.7)	0.94	0.99 (0.74-1.33)
AA	48(25.8)	38(22.5)						
rs2272522								
CC	78(41.9)	82(48.5)		C	236(63.4)	240(71.0)		C *vs*. T
CT	80(43.0)	76(45.0)	0.03	T	136(36.6)	98(29.0)	0.03	0.71 (0.52-0.97)
TT	28(15.1)	11(6.5)						
rs2055314								
CC	59(31.7)	34(20.1)		C	199(53.5)	150(44.4)		C *vs*. T
CT	81(43.5)	82(48.5)	0.04	T	173(46.5)	188(55.6)	0.02	1.44 (1.07-1.94)
TT	46(24.7)	53(31.4)						
rs10510181								
GG	57(30.6)	36(21.3)		G	199(53.5)	152(45.0)		G *vs*. A
GA	85(45.7)	80(47.3)	0.08	A	173(46.5)	186(55.0)	0.02	1.41 (1.05-1.89)
AA	44(23.7)	53(31.4)						

SNP: single nucleotide polymorphism; AIS: adolescent idiopathic scoliosis.

OR: odds ratio, CI: confidence interval.

**Table 4:** Pooled results of 3 SNPs in and around CHL1 gene in AIS in a Chinese Han population

**Genotype**	**AIS (%)**	**Control (%)**	**P-value (χ2)**	**Alleles**	**AIS (%)**	**Control (%)**	**P-value (χ2)**	**OR (95% CI)**
rs2272522								
CC	140(28.0)	110(22.0)		C	518(51.8)	481(48.1)		C *vs*. T
CT	238(47.6)	261(52.2)	0.08	T	482(48.2)	519(51.9)	0.09	1.16 (0.97-1.38)
TT	122(24.4)	129(25.8)						
rs2055314								
CC	217(43.4)	220(44.0)		C	670(67.0)	663(66.3)		C *vs*. T
CT	236(47.2)	223(44.6)	0.50	T	330(33.0)	337(33.7)	0.74	1.03 (0.86-1.24)
TT	47(9.4)	57(11.4)						
rs10510181								
GG	144(28.8)	149(29.8)		G	519(51.9)	542(54.2)		G *vs*. A
GA	231(46.2)	244(48.8)	0.39	A	481(48.1)	458(45.8)	0.30	0.91 (0.77-1.09)
AA	125(25.0)	107(21.4)						

SNP: single nucleotide polymorphism; AIS: adolescent idiopathic scoliosis.

OR: odds ratio, CI: confidence interval.

In stage I, the genotype frequencies for rs2055314 and rs2272522 were significantly different between case and control groups (P <0.05), and it was marginally different for rs10510181 (P = 0.08). Similarly, the allelic frequencies for rs2055314, rs2272522, and rs10510181 showed significant differences between case and control groups (P <0.05) (Table 3). Otherwise, the genotype and allelic frequencies for the other two SNPs (rs331894 and rs2272524) were comparable between case and control groups (P >0.05) (Table 3).

In stage II, the sample size was enlarged for rs2055314, rs2272522, and rs10510181. The pooled data were shown in Table 4. For rs10510181, the genotype frequencies were GG 28.8%, GA 46.2%, and AA 25.0% in AIS patients and GG 29.8%, GA 48.8%, and AA 21.4% in controls. No significant difference was found in genotype distribution between cases and controls (P = 0.39). In addition, there was no obvious difference in allelic distribution between the two groups (G: 51.9% in cases vs. 54.2% in controls, A: 48.1% in cases vs. 45.8% in controls, P = 0.30). Similarly, the genotype and allelic distributions for rs2055314 and rs2272522 were comparable between case and control groups (Table 4).

## Discussion

It is generally recognized that abnormal growth is associated with the development and progression of the scoliotic curves. After the onset of puberty, significantly longer corrected height, arm span, and various body segments were found. Furthermore, BMI of AIS was significantly lower than that of controls [19]. In the present study, we also found that the body weight was significantly lower in AlS patients than in normal controls.

GWAS is a powerful method for the detection of genetic contributions to polygenic diseases, and it has been used to identify genetic predisposition in AIS, which has been recently regarded as one of the most common complex genetic disorders of the musculoskeletal system [6,15]. However, this method may produce spurious associations, as the findings could be confounded by systematic biases in comparison groups, such as population stratification and effect heterogeneity [20,21]. Therefore, replicating the associations in different ethnic groups, as well as studies with large sample sizes, is important to confirm the results of GWAS [22]. Recently, Sharma et al. [15] reported the association between SNP rs10510181, in the proximity of the CHL1 gene, and AIS by performing GWAS in 419 Caucasian families with AIS. The present study was conducted to validate the association of SNP rs10510181 with AIS. The results showed that rs10510181 was not associated with AIS in a Han Chinese population. Furthermore, 4 SNPs covering the entire region of the CHL1 gene were not associated with the occurrence of AIS either, indicating that the CHL1 gene might not be associated with AIS.

The previously reported association of rs10510181 with AIS predisposition in Caucasians was not replicated in our study. Several factors could lead to this lack of replication, which is not rare in genetic association studies of common diseases. First, the allele and genotype frequencies of rs10510181 could vary with ethnicity. Because of differences in variant frequency and underlying linkage disequilibrium (LD) structure between ancestral populations, the relationship between rs10510181 and a pathogenic variant may be influenced. Second, the inclusion criteria were different between the two studies. The subjects included in Sharma's study [15] had a curvature larger than 10 degrees, as well as axial rotation toward the side of the deviation. In the present study, patients with a Cobb angle greater than 20 degrees were included.

The CHL1 gene encodes an axon guidance protein that plays an important role in the guidance of thalamocortical axons [23], as well as the proliferation and differentiation of neural progenitor cells [24]. Mutations in the CHL1 gene may alter the axonal guidance and brain anatomy in mice [25]. Moreover, mutations in the Robo3 gene, which encodes an axon guidance protein related to *CHL1*, could cause HGPPS [17]. Among the hypotheses of AIS etiology, abnormalities in the central nervous system have long been thought to play a key role [26,27]. Disturbance of the central nervous system may impair somatosensory function and motor adaptation, which lead to the asymmetry of neuromuscular condition causing AIS [27,28]. Thus, it seems quite plausible that *CHL1* could be involved in the etiology of scoliosis through disturbance of the central nervous system. Although the present study did not find any association between the four SNPs spanning the whole region of the CHL1 gene and AIS, we cannot entirely exclude the role of this gene in the development of AIS because the CHL1 gene spans more than 100 kb and contains thousands of SNPs.

## Conclusions

The current study did not find an association between the SNPs in and around the CHL1 gene and AIS predisposition. Considering the power calculation and sample size of the present study, we conclude that there is no association between the CHL1 gene and AIS predisposition in a Han Chinese population. However, the role of the CHL1 gene in other populations cannot be excluded, and replication studies in other ethnic groups are needed to understand the overall significance of the CHL1 gene in AIS.

## Competing interests

The authors declare that they have no competing interests.

## Authors’ contributions

XSQ and FL performed the genetic studies. ZZZ and BPQ recruited patients and were involved in the clinical part of the investigation. BW performed the statistical analysis. YQ conceived the study, participated in its design and coordination and helped to draft the manuscript. All of the authors read and approved the final manuscript.

## Pre-publication history

The pre-publication history for this paper can be accessed here:

http://www.biomedcentral.com/1471-2474/15/38/prepub
